# Antibody-Based Targeting of Interferon-Beta-1a Mutein in HER2-Positive Cancer Enhances Antitumor Effects Through Immune Responses and Direct Cell Killing

**DOI:** 10.3389/fphar.2020.608774

**Published:** 2021-01-08

**Authors:** Chan Gyu Lee, TaeEun Kim, Sungyoul Hong, Jongwan Chu, Ju Eun Kang, Hee Geon Park, Jun Young Choi, Kyoung Song, Sun Young Rha, Soohyeon Lee, Joon-Seok Choi, Sun Min Kim, Hae Min Jeong, Young Kee Shin

**Affiliations:** ^1^ Laboratory of Molecular Pathology and Cancer Genomics, College of Pharmacy, Seoul National University, Seoul, South Korea; ^2^ Genopharm Inc., Seoul, South Korea; ^3^ Department of Molecular Medicine and Biopharmaceutical Sciences, Graduate School of Convergence Science and Technology, Seoul National University, Seoul, South Korea; ^4^ R&D Center, ABION Inc., Seoul, South Korea; ^5^ College of Pharmacy, Duksung Women’s University, Seoul, South Korea; ^6^ Yonsei Cancer Center, Yonsei University College of Medicine, Seoul, South Korea; ^7^ Division of Oncology-Hematology, Department of Internal Medicine, Korea University College of Medicine, Seoul, South Korea; ^8^ College of Pharmacy, Daegu Catholic University, Gyeongsan, South Korea; ^9^ Department of Obstetrics and Gynecology, Seoul Metropolitan Government Seoul National University Boramae Medical Center, and Seoul National University College of Medicine, Seoul, South Korea; ^10^ Research Institute of Pharmaceutical Sciences, College of Pharmacy, Seoul National University, Seoul, South Korea; ^11^ Bio-MAX/N-Bio, Seoul National University, Seoul, South Korea

**Keywords:** HER2, trastuzumab, interferon-beta mutein, immunocytokine, tumor-targeting, immune cell-mediated cytotoxicity

## Abstract

Type I interferon (IFN) has been approved as an anticancer agent to treat some malignancies. However, IFNs have a short *in vivo* half-life, systemic toxicity, and poor biophysical properties, which prevent it from being widely used for cancer therapy. This study aimed to construct recombinant IFN-β-1a mutein immunocytokines that comprise a human epidermal growth factor receptor 2 (HER2)-targeting antibody and IFN-β muteins with an additional glycosylation, which can overcome the limitation of the cytokine itself. Hence, the molecular design aims to 1) enhance productivity and biophysical properties by adding secondary glycosylation in IFN-β, 2) increase the therapeutic index of IFN-β therapy by preferential retention at the tumor by possessing high affinity for HER2-expressing cancer cells, and 3) improve the pharmacokinetics and, thus, the convenience of IFN-β administration. The yield of trastuzumab-IFN-β mutein was higher than that of trastuzumab-wild-type IFN-β in the mammalian cell culture system. Trastuzumab-IFN-β mutein showed similar IFN activity and HER2-targeting ability equivalent to that of IFN-β mutein and trastuzumab, respectively. Trastuzumab-IFN-β mutein directly inhibited the growth of HER2-positive gastric cancer cell lines and was more effective than trastuzumab or IFN-β mutein alone. Trastuzumab-IFN-β mutein and IFN-β mutein displayed enhanced immune cell-mediated cytotoxicity. Collectively, trastuzumab-IFN-β mutein may have indirect immune cell-mediated antitumor effects and direct cell growth inhibitory effects. Tumor-targeting effect of trastuzumab-IFN-β mutein was analyzed using *in vivo* fluorescence imaging. The accumulation of trastuzumab-IFN-β mutein was observed in HER2-positive tumors rather than other tissues except the liver. To evaluate the both direct tumor growth inhibition effect and indirect immune cell-mediated antitumor effect, we tested the effect of trastuzumab-IFN-β mutein in HER2-positive cancer xenograft models using nude mice or humanized mice. Trastuzumab-IFN-β mutein could significantly enhance tumor regression when compared with trastuzumab or IFN-β mutein. In addition, an increase in tumor-infiltrating lymphocytes was observed in the trastuzumab-IFN-β mutein-treated group, implying that the tumor-targeting IFN-β may have an enhanced antitumor effect through increased immune response. Therefore, targeting IFN-β with an anti-HER2 monoclonal antibody makes the immunocytokine more potent than either agent alone. These novel findings suggest that trastuzumab-IFN-β mutein merits clinical evaluation as a new candidate of anticancer therapeutics.

## Introduction

During the last decade, the pleiotropic antitumor effects of type I interferons (IFNs) have been studied, with specific reference to their direct role on cancer cells and indirect action through the immune effector cells and tumor vasculature (Borden, 2019). Based on the antitumor efficacy, a recombinant human type I IFN, IFN-α2, has been approved for the treatment of different types of cancers (Borden et al., 2007). However, systemic administration of IFNs has severe technical limitations that must be overcome. This mainly includes the failure to achieve optimal concentrations within the tumor bed after administration of tolerable dose of IFNs. IFNs have short half-lives (Radwanski et al., 1987; Salmon et al., 1996), and pharmacokinetic studies have shown that only a tiny fraction of the injected dose (approximately 0.01%) reaches the targeted tumor regions and draining lymph nodes (Suzuki et al., 2003). Since the potential of cytokine therapies are impaired due to dose-limiting systemic toxicities, novel treatment methods should be developed to safely deliver effective drug quantities at the tumor sites relative to the whole body (Young et al., 2014).

Antibody-drug conjugates (ADCs) has been attempted to induce organ-targeting and alleviate systemic side effects, and several ADCs such as trastuzumab emtansine (T-DM1) have been successfully approved (Hoffmann et al., 2018). When conjugated with an antibody, higher doses of payload can be tolerated (Poon et al., 2013). Immunocytokines share a similar development concept, so it is predicted that they could improve the therapeutic window of cytokines. In cases of IFNs, various conjugates with monoclonal antibody (mAb) targeted against tumor-associated proteins, such as EGFR, HER2, CD20, CD38, and CD137, showed antiproliferative effects in murine models of either hematological malignancies or solid tumors (Huang et al., 2007; Rossi et al., 2009; Dubrot et al., 2011; Trinh et al., 2013; Yang et al., 2014; Vega et al., 2015; Pogue et al., 2016). Tumor suppression was mediated by IFN α/β receptor on the cells (Xuan et al., 2010) and/or through a more effective tumor antigen presentation by DCs to CD8^+^ T cells (Yang et al., 2014). In addition, therapeutic advantages of IFN-antibody conjugates might be derived through immunomodulation and angiogenesis blockade (Greiner et al., 1991; Ozzello et al., 1995; Sivaraman et al., 2000; Li et al., 2017).

The uncertainty of IFN’s inter-species reactivity discourages the development of human type I IFN derivatives for clinical application. The limited cross-reactivity is probably due to low cross-species identification (Harari et al., 2014). Cytokines including type I IFNs, IL-2, IL-7, IL-15, and TNFα, attract attention as anticancer agents (Conlon et al., 2019), and their receptors have 50–80% homologies between humans and mice. In particular, type I IFN receptors and IFN-β had the least homology (less than 50%), and as a result, other cytokines had some cross-reactivity, whereas IFN- β did not (Mosmann et al., 1987; Seckinger et al., 1990; Eisenman et al., 2002; Barata et al., 2006). Human to mouse cross-reactivity is a very important factor, especially in conducting *in vivo* experiments. Because it is very difficult to evaluate the full efficacy and toxicity of human IFN-β substrates to host cells in murine models, most preclinical studies used mouse IFN surrogates to analyze the *in vivo* effect, and revealed the underlying mechanisms. Nevertheless, for clinically relevant studies, human form of IFN substrates should be tested.

In our previous studies, we developed a glycoengineered variant of recombinant human IFN-β-1a, IFN-β-R27T, which has two N-glycosylation sites at the 80th (original site) and an additional one at the 25th amino acid residue due to a mutation of Thr to Arg at position 27 of IFN-β (Song et al., 2014). IFN-β-R27T exhibited superior stability, solubility, productivity, and pharmacokinetic properties compared to wild-type IFN-β-1a (Lee et al., 2019; Song et al., 2020). Herein, we constructed fusion proteins consisting of an anti-HER2 antibody (trastuzumab) with IFN-β mutein and investigated its antitumor effect on a HER2-positive model.

## Materials and Methods

### Antibody and Antibody-Cytokine Fusion Proteins

The trastuzumab- and trastuzumab-IFN-β mutein-expressing gene constructs were generated by gene synthesis (Cosmogenetech, Seoul, Korea), and the synthesized heavy and light chain DNAs were inserted into the pCHO 1.0 expression vector (Life Technologies, Gaithersburg, MD, United States) at the AvrII-Bstz17I and EcoRV-PacI sites of the polylinker region, respectively (Supplementary Table S1). CHO-S cells (Life Technologies, Gaithersburg, MD, United States) were transfected with the expression vectors, and stable clones were selected with 100–10,000 nM of methotrexate (Sigma, NY, United States) and 10–50 μg/ml of puromycin (Life Technologies, Gaithersburg, MD, United States). Culture media from CHO-S cells stably expressing trastuzumab and trastuzumab-IFN-β muteins were collected and loaded onto a MabSelect SuRe^TM^ rProtein A agarose-bead resin (GE Healthcare, WI, United States), and the proteins were eluted using 0.1 M sodium citrate buffer (pH 3.0). The purified antibody and antibody-cytokine fusion proteins were quantified using the Cedex Bio Analyzer (Roche, Indianapolis, IN, United States) and analyzed using sodium dodecyl sulfate-polyacrylamide gel electrophoresis (SDS-PAGE) under reducing and non-reducing conditions.

### Cell Lines and Cell Culture

Human gastric carcinoma cell lines (NCI-N87, YCC-19, YCC-38, KATOIII, Hs746T, and MKN74), normal gastric epithelial cell line (HFE145), and normal primary human umbilical vein endothelial cell line (HUVEC) were used in this study. NCI-N87 and HUVEC cell lines were purchased from the American Type Culture Collection (ATCC; Manassas, VA, United States). KATOIII, Hs746T, and MKN74 cell lines were purchased from Korean Cell Line Bank (KCLB; Seoul, Korea). YCC-19 and YCC-38 cell lines were established and provided by Sun Young Rha (Yensei University, Seoul, Korea) (Kim et al., 2018). HFE145 cell line was provided by Hassan Ashktorab (Howard University, MD, United States) (Marlink et al., 2003). All cell lines were cultured with RPMI 1640 Medium (Hyclone, Logan, UT, United States) supplemented with 10% fetal bovine serum (FBS) (Hyclone, Logan, UT, United States), 100 units/ml penicillin, and 100 μg/ml streptomycin (Hyclone, Logan, UT, United States). HUVECs were maintained in VascuLife EnGS (containing endothelial cell growth supplement; Lifeline Cell Technology, Frederick, MD, United States). All cultured cells were incubated at 37°C in a cell culture incubator with 5% CO_2_.

### Three-Dimensional (3D) Structure, Circular Dichroism, and Size Analysis

The 3D structure of trastuzumab-IFN-β was predicted using the Protein Data Bank (PDB) files of trastuzumab and IFN- β-1a. To determine the secondary structures of trastuzumab-IFN-β-R27T (trastuzumab-R27T), circular dichroism spectra were obtained using a Chirascan-plus circular dichroism spectrometer (Applied Photophysics Ltd., Surrey, United Kingdom). The spectra were measured between the wavelengths of 190 and 260 nm in the presence of samples diluted in phosphate-buffered saline (PBS). The secondary structure of the trastuzumab-R27T was analyzed using CDNN secondary structure analysis software (Applied Photophysics Ltd., Surrey, United Kingdom). The size of trastuzumab-R27T was measured using dynamic light scattering.

### Flow Cytometry Analysis

To analyze the HER2 binding of trastuzumab and trastuzumab-R27T, NCI-N87 and HUVEC cells were detached with enzyme-free, PBS-based cell dissociation buffer (Gibco, Gaithersburg, MD, United States). The cells were incubated with 1 μg/ml of human immunoglobulin G1 (IgG) (Jackson Immunoresearch Laboratories, West Grove, PA, United States), trastuzumab or trastuzumab-R27T in PBS containing 1% FBS for 1 h at 4°C. The cells were then washed twice and incubated with fluorescein isothiocyanate (FITC)-conjugated anti-human secondary antibody (diluted 1:100; Santa Cruz Biotechnology, Danvers, MA, United States). Stained cells were analyzed using a BD FACSCalibur system equipped with Cell Quest Pro software (BD Biosciences, San Jose, CA, United States).

### Immunofluorescence Analysis

NCI-N87 and HUVECs were seeded in four-well culture slides (SPL, Seoul, Korea), grown to 80% confluence and treated with trastuzumab or trastuzumab-R27T for 1 h. The cell culture slides were washed with PBS, and cells were fixed using 4% paraformaldehyde for 15 min. The cells were incubated with FITC-conjugated anti-human secondary antibody (Santa Cruz, Danvers, MA, United States) for 1 h and stained with 4,6-diamidino-2-phonylindole dihydrochloride. The slides were removed from chamber and mounted for digital micrographs, which were taken using the LSM 700 ZEISS laser scanning confocal microscope (Carl Zeiss, Jena, Germany). The data were processed using LSM co-localization software (Carl Zeiss, Jena, Germany).

### IFN Luciferase Assay

IFN reporter gene assays were performed using iLite^®^ Type I IFN assay ready cells (Euro Diagnostica, Malmo, Sweden) and a luciferase assay kit (Promega, Madison, WI, United States). Briefly, iLite^®^ Type I IFN assay ready cells were seeded in a 96-well plate and treated with IFN-β-R27T, trastuzumab, or trastuzumab-R27T. After 18 h, type I IFN promoter activity was quantified by measuring the firefly luciferase luminescence using a microplate reader (TECAN, Männedorf, Switzerland).

### Cell Viability Assay

To perform the cell viability assay, NCI-N87, YCC-19, YCC-38, KATOIII, Hs746T, MKN74, or HFE145 cells were seeded in 96-well plates, cultured overnight, and treated with various concentrations of IFN-β-R27T, trastuzumab, or trastuzumab-R27T for 72 h. Cell viability was assessed by Water-Soluble Tetrazolium (WST) colorimetric assay (Ez-Cytox; DogenBio, Seoul, Korea). Absorbance was measured at 450 nm using a microplate reader (TECAN, Männedorf, Switzerland). Percent growth was calculated according to the NCI-60 DTP Human Tumor Cell Line Screen protocol as follows:

% Growth = (treated viability – viability at time 0)/(vehicle control viability – viability at time 0) × 100.

Growth inhibition of 50% (GI_50_) and the maximum inhibitory effect at the highest drug concentration (E_max_) values were estimated using GraphPad Prism 7.0 software (San Diego, CA, United States).

### Immune Cell-Mediated Cytotoxicity Assay

Human peripheral blood mononuclear cells (PBMCs) were purchased from Stem Express (Placerville, CA, United States). CD14^+^ monocytes, CD8^+^ T cells, CD4^+^ T cells, or natural killer (NK) cells were purified from human PBMCs using magnetic-activated cell sorting (Miltenyi Biotech GmbH, Bergisch Gladbach, Germany) according to the manufacturer’s instructions. To perform the immune cell-mediated cytotoxic assay, the human gastric cancer cells were seeded at 1 × 10^4^ cells per well in 96-well plates. The next day, tumor cells were co-cultured with each effector cell population at 0.5:1, 1:1, 2:1, 4:1, 8:1, and 16:1 effector-to-target cell (E:T) ratios in the absence or presence of control IgG, trastuzumab, IFN-β-R27T, or trastuzumab-R27T for 72 h. After incubation, cell viability was assessed by WST colorimetric assay (DoGenBio, Seoul, Korea). Absorbance at wavelength 450 nm was read using a microplate reader (TECAN, Männedorf, Switzerland), and data were recorded and normalized by respective blank using target and effector cell culture. In the wash-out study, NCI-N87 or Hs746T cells were pre-treated with control IgG, trastuzumab, IFN-β-R27T, or trastuzumab before co-culture with PBMC. After 6 h, the cells were washed with PBS twice and then co-cultured with PBMCs.

### Antibody-Dependent Cell-Mediated Cytotoxicity Assay

The ADCC assay was performed in four human gastric cancer cell lines (NCI-N87, YCC-19, KATOIII, and Hs746T) and two normal cell lines (HFE145 and HUVEC). Target cells were seeded in 96-well plates at a density of 2 × 10^4^ cells/well and incubated overnight. The NK-92MI-CD16a cells, which stably express CD16a, were previously established and used as effector cells (Yang et al., 2020). The target cells were incubated with control IgG, IFN-β-R27T, or trastuzumab-R27T at a final concentration of up to 10,000 nM and 8 × 10^4^ effector cells in a CO_2_ incubator for 4 h at 37°C (E:T ratio = 4:1). Target cell lysis was measured by detecting the release of lactate dehydrogenase using CytoTox 96^®^ Non-Radioactive Cytotoxicity Assay (Promega, Madison, WI, United States) according to the manufacturer’s instructions. The absorbance of the plates was analyzed using the Spark TM 10M microplate reader (TECAN, Männedorf, Switzerland) at 490 nm. For data analysis, the percentage of specific ADCC was calculated as follows:

% Cytotoxicity = (Experimental – Effector Spontaneous – Target Spontaneous)/(Target Maximum – Target Spontaneous) × 100.

The dose–response curve and EC_50_ values were estimated using GraphPad Prism 7 (GraphPad Software, San Diego, CA, United States).

### Tumor-Targeting Analysis in a HER-2-Positive Tumor Xenograft Mouse Model

Control IgG-IFN-β-C17S/R27T (IgG-C17S/R27T) and trastuzumab-IFN-β-C17S/R27T (trastuzumab-C17S/R27T) were labeled with fluorescent dye CF750 (Biotium Inc., Hayward, CA, United States) through amide linkage in basic condition in accordance with the manufacturer’s instructions. Since IFN-β-R27T is susceptible to pH stress (Song et al., 2020), C17S was additionally addressed to reduce nonspecific events that occur during labeling. C17S mutation increases stability without affecting IFN-β activity (Runkel et al., 1998), allowing it to generate more stable and high yield products while maintaining the properties of IFN-β-R27T (Supplementary Figure S1).

To generate xenografts, 5 × 10^6^ NCI-N87 or Hs746T cells were resuspended in 100 µL PBS and injected subcutaneously into the right flank of six-week-old BALB/c-nude mice (Orient Bio, Seongnam, Gyeonggi, Korea). Tumor-bearing mice were treated intravenously with CF750-labeled antibody-cytokine fusion proteins (100 µg/100 µL in PBS). After 1, 6, and 24 h, the mice were anesthetized with Terrell TM isoflurane (Piramal Critical Care Inc., Bethlehem, PA, United States) and placed in Ami HTX (Spectral Instruments Imaging, Tucson, AZ, United States) to visualize CF750-labeled antibody-cytokine fusion proteins. The fluorescence was detected using an excitation filter (710 nm) and an emission filter (790 nm). At the final time point, the mice were sacrificed, and the liver, kidney, spleen, lung, intestine, and tumor were excised. The organs from each mouse were placed, and the intensity of fluorescence was analyzed using Aura imaging software (Spectral Instruments Imaging, Arizona, United States). The average fluorescence intensity was calculated by creating a region of interest for each organ. The animal experiment was approved by the Institutional Animal Care and Use Committee (IACUC) of Seoul National University (SNU-200307-1).

### Human Gastric Cancer Xenograft Nude Mice Model

The antitumor efficacy of trastuzumab-IFN-β mutein *in vivo* was tested in NCI-N87 xenograft models. The animal experiment was approved by the Institutional Animal Care and Use Committee (IACUC) of Seoul National University (SNU-190528-2-1). Five-week-old male/female BALB/c-nude mice (Orient Bio, Seongnam, Gyeonggi, South Korea) were subcutaneously inoculated at the dorsal right side with 5 × 10^6^ cells. NCI-N87-bearing mice were intraperitoneally treated with trastuzumab, IFN-β-R27T, or trastuzumab-R27T (10 mg/kg) thrice a week for 3 weeks. Tumor size was measured using a Vernier caliper in two dimensions, and the tumor volume was calculated using the following formula: tumor volume (mm^3^) = (short diameter)^2^ × (long diameter) × 0.5.

### Humanized Gastric Cancer Xenograft in Humanized Mice

To establish humanized mice, mononuclear cells were separated from human umbilical cord blood obtained after normal full-term deliveries. Informed consent was obtained from donor according to institution guidelines, and these experiments were approved by Seoul Metropolitan Government Seoul National University Boramae Medical Center (IRB No. 16-2014-80) and Seoul National University Institutional Review Board (IRB No. E1409/002-001). This study is conducted in accordance with the World Medical Associtation’s Declaration of Helsinki. CD34^+^ hematopoietic stem cells (HSCs) were isolated using a direct CD34^+^ MicroBead kit (Miltenyi Biotec, CA, United States). Five-week-old female NSG mice (Jackson Laboratory, PA United States) were preconditioned with 30 mg/kg of busulfan (MedChemExpress, Monmouth Junction, NJ, United States). Approximately, over 90% purified 1 × 10^5^ of freshly isolated CD34^+^ HSCs were injected intravenously into mice 24 h after preconditioning. The engraftment levels of human CD45^+^ cells and human immune cell populations were determined in the peripheral blood using a color flow cytometry panel. Mice that had over 25% human CD45^+^ cells in the peripheral blood were considered humanized. Humanized mice were randomized into every treatment group for all experiments. All humanized mice were confirmed for humanization before tumor xenograft.

To generate subcutaneous xenograft tumor models, 1 × 10^6^ NCI-N87 or YCC-19 cells were implanted in the right flank of humanized mice. Tumor-bearing humanized mice were randomized into the treatment and non-treatment groups. Three humanized mice per group, from one donor, were used for xenograft experiments. Tumor-bearing humanized mice were treated with trastuzumab, IFN-β-R27T, or trastuzumab-R27T intraperitoneally thrice a week for 3 weeks. The control group received intraperitoneal injections of the saline vehicle. Mice were monitored routinely, and tumor size was measured with calipers at the endpoint, and volumes (in mm^3^) were calculated using the formula (length × width^2^)/2. For immune analysis, mice from each treatment groups were sacrificed, and tumor tissues were harvested for IHC. Harvested tissues were fixed with 4% paraformaldehyde for 24 h and paraffin-embedded for immunostaining with the indicated antibodies. Human forkhead box P3 (FOXP3; clone D9M8I), CD68 (clone D4B9C), and programmed death-ligand 1 (PD-L1; clone E1L3N) antibodies were purchased from Cell Signaling Technology (United States), and human CD8 antibody was purchased from Invitrogen (United States). Histology slides were analyzed with a photomicroscope (Olympus, Miami, FL, United States) at a final magnification of 200×. Six images from each group were captured using NIS-Element BR software (Nikon, Shizuoka, Japan).

### Statistical Analysis

Data were presented as means ± standard deviation (SD) and were statistically analyzed using one or two-way analysis of variance with appropriate post hoc analysis for multiple groups or student’s unpaired two-tailed t-tests. A *p* value less that 0.05 was considered statistically significant. All statistical analyses were performed using GraphPad Prism 7.0 software (San Diego, CA, United States).

## Results

### Generation of Trastuzumab-IFN-β Mutein Fusion Protein

**FIGURE 1:**
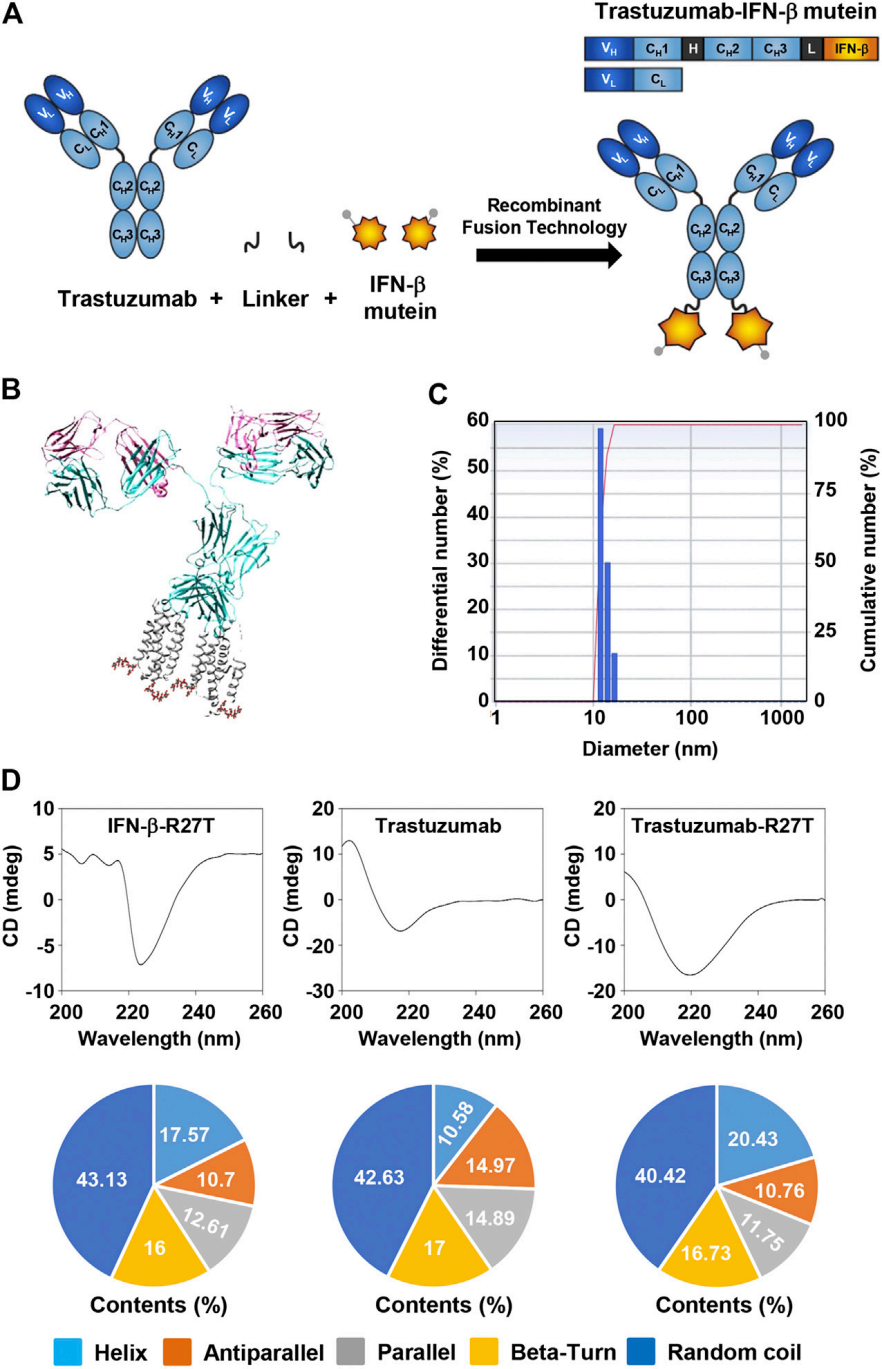
Generation of trastuzumab-IFN-β mutein fusion protein. **(A)** Schematic representation of trastuzumab-IFN-β mutein and its constituents. The fusion protein was generated by fusing IFN-β mutein to the C-terminus of the trastuzumab heavy chain via 15-mer flexible peptide linker (GGGGSGGGGSGGGSG). **(B)** A model structure of trastuzumab-IFN-β. 3D structure of trastuzumab-IFN-β was predicted *in silico*. **(C)** The size of trastuzumab-IFN-β was measured by dynamic light scattering. **(D)** Structural analysis of trastuzumab-R27T. The CD spectra of IFN-β-R27T, trastuzumab, and trastuzumab-R27T were measured. The secondary structural contents were analyzed using the CDNN program.

We generated an anti-HER2 antibody conjugated IFN-β-R27T fusion protein (trastuzumab-R27T), in which a 15-mer flexible peptide linker (GGGGSGGGGSGGGSG) was used to link IFN-β-R27T to the C-terminus of each heavy chain of trastuzumab (Figure 1A). Trastuzumab-R27T was expressed in Chinese hamster ovary (CHO) cells and secreted in the culture medium. The proteins were purified and separated by SDS-PAGE under reducing and non-reducing conditions. The band representing the heavy chain of the fusion protein was observed between the 70 and 100 kDa marker bands, which confirmed that IFN-β-R27T was successfully fused to the heavy chain (Supplementary Figure S2A). The fusion protein showed a higher molecular weight than the trastuzumab band under non-reducing conditions (Supplementary Figure S2B). Next, we compared the expression levels of trastuzumab-wild-type IFN-β and trastuzumab-R27T fusion proteins in CHO-S cells. Expression analysis revealed that trastuzumab-R27T showed higher expression levels than trastuzumab-wild-type IFN-β (Supplementary Figure S2C). In addition, IgG quantification analysis revealed that the expression level of trastuzumab-R27T was six-fold higher than that of trastuzumab-wild-type IFN-β (Supplementary Figure S2D). The results are consistent with our previous research on IFN-β-R27T that showed six-fold higher productivity compared to wild-type IFN-β (Song et al., 2014). To summarize, these results suggest that trastuzumab-R27T is appropriately conjugated and produced more stably and efficiently.

The 3D structure of trastuzumab-R27T was visualized using the PDB files for trastuzumab and IFN-β-1a (Figure 1B). The size of trastuzumab-R27T was approximately 16.8 nm, which corresponds to the combined size of IFN-β-R27T (3.7 nm) and trastuzumab (11.4 nm) (Figure 1C). Moreover, the secondary structural analysis revealed that the trastuzumab-R27T tends to form α-helices that are 9.85% higher than that of trastuzumab, mainly due to the conjugation of IFN-β (Figure 1D).

### Bioactivity of the Trastuzumab-IFN-β Mutein Fusion Protein

**FIGURE 2:**
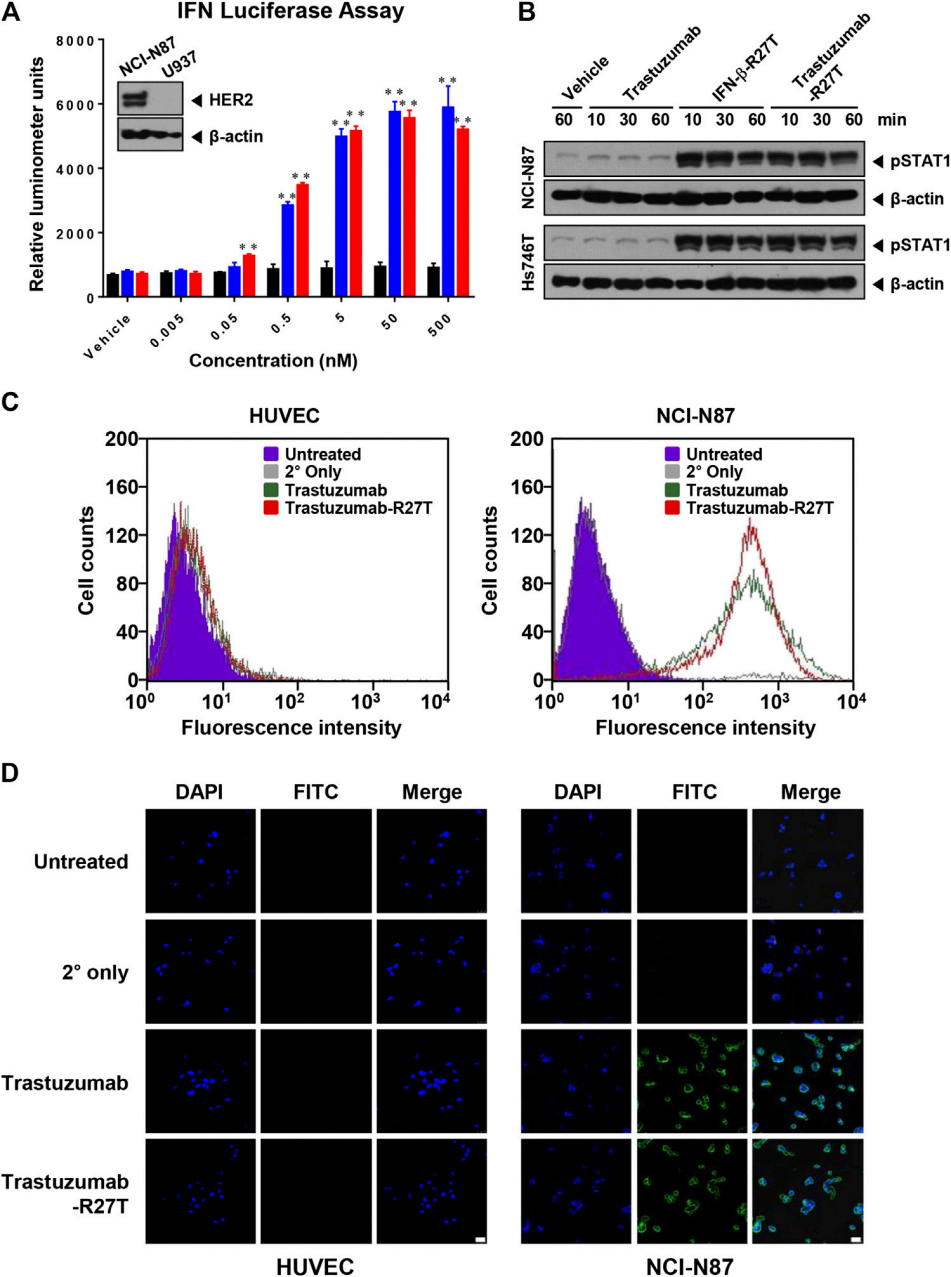
Anti-HER2 and IFN-β bioactivity of trastuzumab-IFN-β mutein. **(A)** Type I IFN function of trastuzumab-R27T. U937 cells stably expressing an ISG promoter-firefly gene were treated with or without the indicated concentration of trastuzumab, IFN-β-R27T, or trastuzumab-R27T. After 18 h, the firefly luciferase activities were determined. **(B)** STAT1 phosphorylation of trastuzumab-R27T. NCI-N87 or Hs746T cells were treated with or without trastuzumab, IFN-β-R27T, or trastuzumab-R27T for the indicated times. The cell lysates were analyzed by western blotting with the indicated antibodies. **(C)** HER2 binding assay. HUVECs and NCI-N87 cells were stained with trastuzumab or trastuzumab-R27T, and fluorescence-positive cells were measured by flow cytometry. **(D)** Analysis of HER2-targeting function by confocal microscopy. HUVECs and NCI-N87 cells were incubated with trastuzumab, IFN-β-R27T, or trastuzumab-R27T for 1 h, and cellular fluorescence was observed by confocal microscopy. Each bar is the mean of three replicates, and error bars represent standard deviation (SD), **p < 0.01 vs. vehicle control.

To investigate whether the bioactivity of trastuzumab-R27T was maintained, the effects of trastuzumab-R27T on anti-HER2 and IFN activity were evaluated. First, we used the U937 iLite^®^ type I IFN assay ready cell system to confirm the bioactivity of trastuzumab-R27T. This cell line expresses firefly luciferase under the control of IFN-responsive promoter. The result showed that trastuzumab-R27T increased luciferase activity and had similar luminescence as that of IFN-β-R27T (Figure 2A). Since U937 iLite^®^ ready cells do not express HER2 (Figure 2A, insert), the IFN activation in the cells induced by trastuzumab-R27T was independent of HER2 expression. The IFN signaling is mediated through the binding of type I IFN receptors and the subsequent phosphorylation of signal transducers and activators of transcription (STATs) (Darnell et al., 1994). Trastuzumab-R27T induced robust STAT1 phosphorylation in both NCI-N87 and Hs746T gastric cancer cells harboring high and low HER2 expression, respectively (Figure 2B). These results indicate that the cytokine attached to the C-terminal heavy chain of trastuzumab with a polypeptide linker is sterically distant from the antigen-binding region, and is flexible enough to allow engagement with its receptor. Second, to determine the HER2-targeting function of trastuzumab-R27T, HUVEC (HER2-negative) or NCI-N87 cells (HER2-positive) were stained with trastuzumab or the fusion protein. Flow cytometry analysis revealed that trastuzumab and trastuzumab-R27T showed similar binding abilities in both cell lines, indicating that the fusion protein retained the ability to bind to HER2 (Figure 2C). NCI-N87 cell membrane was positively stained by both trastuzumab and fusion protein, whereas HUVECs did not show any other binding (Figure 2D). Overall, the IFN-β mutein-fused trastuzumab possessed both IFN activity and HER2-targeting ability equivalent to that of IFN-β mutein and trastuzumab, respectively.

### *In vitro* Antitumor Effect of the Trastuzumab-IFN-β Mutein Fusion Protein

**FIGURE 3:**
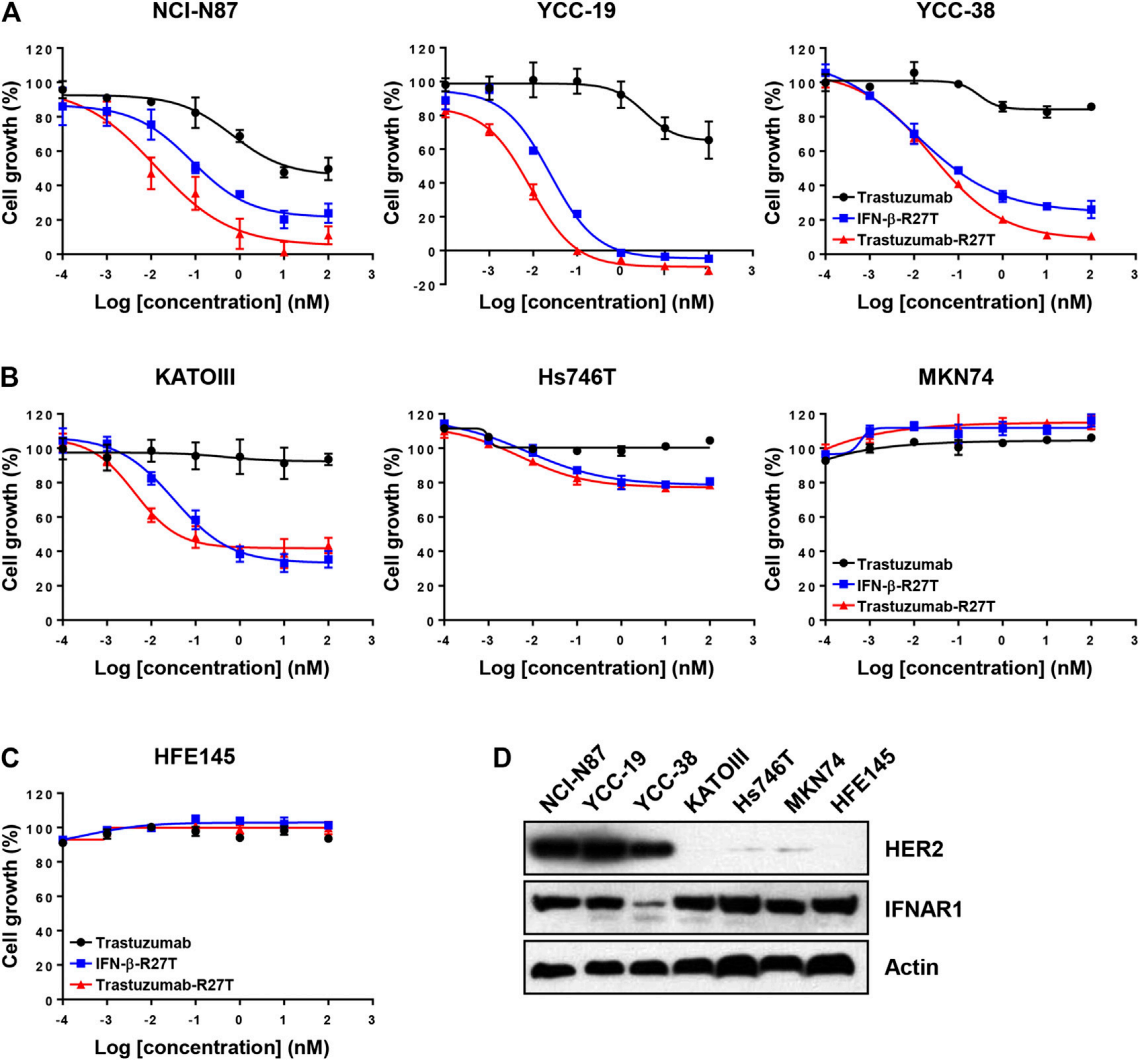
*In vitro* direct antitumor effect of trastuzumab-IFN-β mutein on different gastric cancer cell lines. **(A)** HER2-positive (NCI-N87, YCC-19, and YCC-38), **(B)** HER2-negative (KATOIII, Hs746T, and MKN74) cancer cell lines, or **(C)** normal gastric epithelial cells (HFE145) were treated with trastuzumab, IFN-β-R27T, or trastuzumab-R27T for 72 h. Cell growth (%) was determined using the WST assay. Each point is the mean of three replicates, and error bars represent SD. **(D)** HER2 and Type I IFN receptor 1 expression in gastric cancer cell lines and normal gastric epithelial cell line.

Type I IFNs have a direct antiproliferative or cytotoxic effect on primary malignant cells from patients with multiple myeloma and melanoma (Schiller et al., 1986; Grandér et al., 1993) and a few tumor cell lines, such as WM9, Daudi, and OVCAR3 (Leaman et al., 2003; Kim et al., 2017). We compared the antitumor activity of trastuzumab, IFN-β-R27T, and trastuzumab-R27T using an *in vitro* proliferation assay with HER2-positive (NCI-N87, YCC-19, YCC-38, and SNU216; Figure 3A and Supplementary Figure S3A) and HER2-negative (KATOIII, Hs746T, MKN74, and MKN45; Figure 3B and Supplementary Figure S3B) gastric cancer cell lines and a normal gastric epithelial cell line (HFE145; Figure 3C). The GI_50_ and E_max_ values of trastuzumab, IFN-β-R27T, and trastuzumab-R27T are summarized in Table 1. Trastuzumab-R27T strongly inhibited (E_max_ = 88.9–100%) HER2-positive cancer cells with high potency (GI_50_ = 0.006–0.062 nM); however, trastuzumab slightly inhibited (E_max_ = 14.1–50.4%) HER2-positive cancer cells and had no inhibitory effect on HER2-negative gastric cancer cell lines and the normal gastric epithelial cell line. IFN-β-R27T showed a similar or slightly weak inhibitory effect on cancer cells compared to trastuzumab-R27T, indicating that the antitumor effect of trastuzumab-R27T is mainly due to IFN-β than that of trastuzumab. Interestingly, both IFN-β mutein-containing drugs (trastuzumab-R27T and IFN-β-R27T) had strong antitumor effect on all four HER2-positive cell lines, whereas differential antitumor reactivity was shown in HER2-negative cell lines. Furthermore, the antitumor activity of IFN-β was not correlated with type I IFN receptor expression (Figure 3D).

**TABLE 1:** The ^a^GI_50_ and ^b^E_max_ values of trastuzumab, IFN-β-R27T, and trastuzumab-R27T.

Cell lines	Trastuzumab		IFN-β-R27T		Trastuzumab-R27T	
	GI_50_ (nM)	E_max_ (%)	GI_50_ (nM)	E_max_ (%)	GI_50_ (nM)	E_max_ (%)
NCI-N87	4.38	50.4	0.098	76.1	0.042	88.9
YCC19	>100	34.7	0.034	104.8	0.006	100
YCC38	>100	14.1	0.071	74.0	0.062	89.5
KATOIII	>100	6.5	0.128	64.4	0.018	56.6
Hs746T	>100	0	>100	19.3	>100	21.5
MKN74	>100	0	>100	0	>100	0
HFE145	>100	6.5	>100	0	>100	0.3

a GI_50_: The concentration of drug needed to inhibit cell growth by 50%.

b E_max_: The growth inhibition percent at the highest drug concentration.

### Immune Cell-Mediated Anticancer Effect of the Trastuzumab-IFN-β Mutein Fusion Protein

**FIGURE 4:**
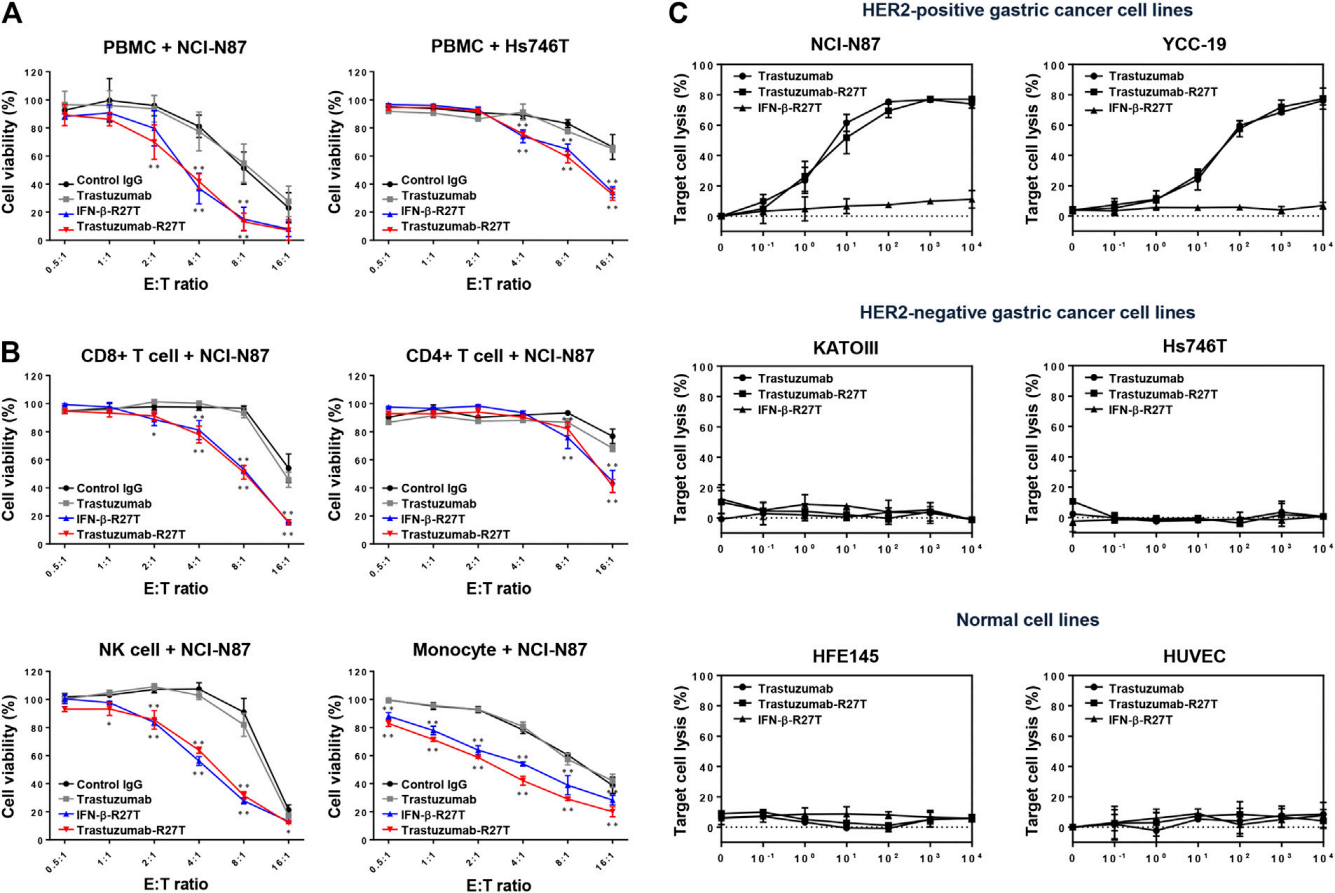
Synergistic effect of trastuzumab-IFN-β mutein on immune cell-mediated cytotoxicity in gastric cancer cell lines. **(A)** PBMCs co-cultured with gastric cancer cells were treated with 0.1 nM of control IgG, trastuzumab, IFN-β-R27T, or trastuzumab-R27T. At 72 h post treatment, cell viability was measured using the WST assay. **(B)** Specific effects of CD8, CD4, NK, and CD14^+^ cells co-cultured with NCI-N87 cells alone or combined with each drug. NCI-N87 cells were co-cultured with sorted CD8^+^ T cells, CD4^+^ T cells, NK cells, and CD14^+^ monocytes at indicated E:T ratio, alone or in combination with each drug. Data represent the mean ± SD (n = 3), *p < 0.05, **p < 0.01 vs. control IgG at each E: T ratio, assessed by two-way ANOVA with Turkey’s multiple comparison test. **(C)** ADCC activity of trastuzumab-R27T in a HER2-dependent manner in various gastric cancer cell lines. Cancer cells were incubated with effector cells at an E:T ratio of 8:1 and various concentration of each drug for 6 h. Cytotoxicity (%) was determined by measuring released lactate dehydrogenase (LDH) and normalizing to a maximum LDH release in the presence of Triton X-100 (100% cell lysis).

Type I IFNs have been proven to be involved in immune system regulation (Hervas-Stubbs et al., 2011). To test whether trastuzumab-IFN-β mutein can activate the antitumor responses of immune cells, we measured PBMC-mediated cytotoxicity in HER2-positive (NCI-N87) or HER2-negative (Hs746T and KATOIII) cells. PBMCs from healthy donors were co-cultured with NCI-N87, Hs746T or KATOIII cells at different E:T ratios ranging from 0.5:1 to 16:1 in the presence of 0.1 nM of human IgG, trastuzumab, IFN-β-R27T, or trastuzumab-R27T. Co-culture with PBMCs exhibited the antitumor function in a certain E:T ratio (Figure 4A; Supplementary Figure S4). Notably, treatment with IFN-β-R27T or trastuzumab-R27T enhanced PBMC-mediated cytotoxicity in all three cell lines, whereas IgG or trastuzumab had no additional effect.

To examine the type of immune cells that contributed to the antitumor effects, the cytotoxic effects of CD4^+^ T cells, CD8^+^ T cells, NK cells, or CD14^+^ monocytes were tested in the NCI-N87 co-culture system. Each cell type was isolated using magnetic bead separation from healthy PBMCs. As shown in Figure 4B, all types of immune cells played a role in restraining NCI-N87 cells. Treatment with IFN-β-R27T or trastuzumab-R27T enhanced the antitumor effect in the presence of each immune cell type. In particular, co-culture with CD8^+^ T cells or NK cells showed the most synergistic cytotoxic effect on NCI-N87 cells in combination with IFN-β-R27T or trastuzumab-R27T. NK cells form a small subset among lymphocytes; however, they are considered the most important cells capable of inducing ADCC in trastuzumab-mediated therapies (Beano et al., 2008). Because activating NK cells rapidly downregulate the expression of Fc gamma receptor III-A (CD16a) (Peruzzi et al., 2013; Romee et al., 2013), co-culture with NK cells from PBMCs did not show sufficient ADCC effect by trastuzumab. To validate the ADCC effect by NK cells, we measured ADCC activity using NK-92MI-CD16 cell line, which is a genetically engineered cell line with sufficient activity (Yang et al., 2020). Treatment of trastuzumab-R27T showed a similar ADCC effect as that of trastuzumab in HER2-positive cancer cell lines, suggesting that trastuzumab-R27T is capable of causing significant ADCC (Figure 4C). These results suggested that the immune response mediated by trastuzumab-IFN-β mutein increased the cytotoxic activity of all types of immune cells, especially the primary effector cells, CD8^+^ T and NK cells, which exerted significant antitumor effects.

### Tumor-Targeting of Trastuzumab-IFN-β Mutein

**FIGURE 5:**
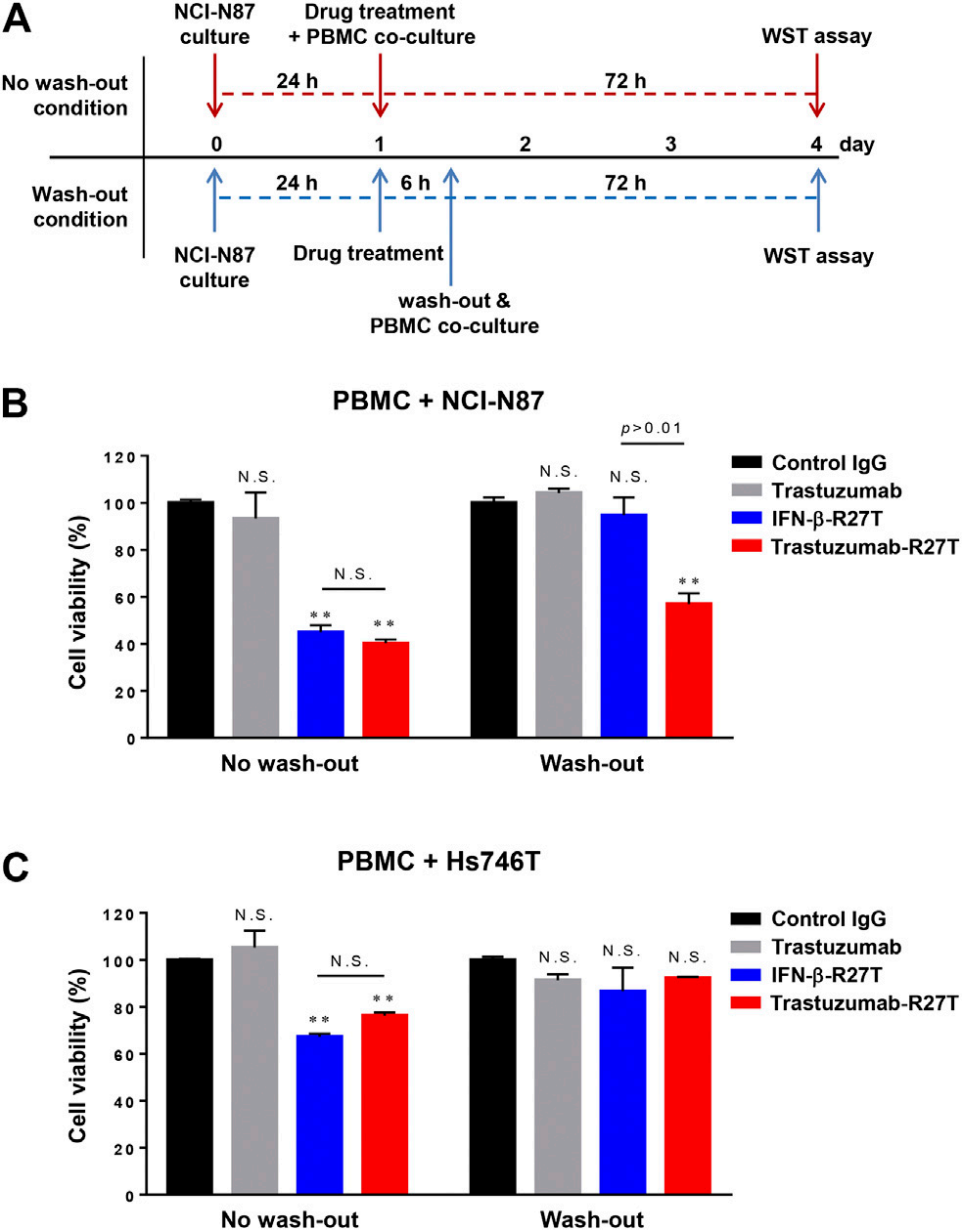
*In vitro* tumor-targeting effect of trastuzumab-IFN-β mutein on immune cell-mediated tumor cytotoxicity. **(A)** Overview of the wash-out study procedure. In the wash-out condition, NCI-N87 cells were pre-treated with each drug before co-culture with PBMCs. After 6 h, cells were washed with PBS twice and then co-cultured with PBMCs at 1:1E:T ratio for 72 h **(B**,**C)** Comparison of anticancer effect of tumor-targeting of IFN-β-R27T in accordance with anti-HER2 antibody fusion in NCI-N87 **(B)** or Hs746T **(C)**. Data represent the mean ± SD (n = 3), **p < 0.01; N.S., not significant.

As shown in Figures 3, 4, IFN-β exhibited both direct cell killing and immune cell-mediated cytotoxicity even in HER2-negative cancer. However, the cytokines that are not targeted to tumors are actually difficult to maintain this effect in an *in vivo* environment. The primary goal of developing immunocytokines is to expand the therapeutic index of IFN-β by preferential retention at the tumor sites by possessing high affinity for antigen-expressing cancer cells. To reveal the importance of the tumor-targeting effect of IFN-β on immune cell-mediated tumor cytotoxicity, we performed a wash-out study in NCI-N87 and Hs746T cells. In the wash-out condition, cancer cells were treated with each drug for 6 h, washed with the completed media, and then co-cultured with PBMCs (Figure 5A). Because treated drugs were intactly remained in the media under non-wash condition, the PBMC-mediated cytotoxic effect of IFN-β-R27T or trastuzumab-R27T was observed in both cell lines (Figures 5B,C). In contrast, drug washing led to the loss of these effects with IFN-β-R27T, while with trastuzumab-R27T they were retained in NCI-N87 cells (Figure 5B right). Although this result was slightly reduced due to the removal of free form of trastuzumab-R27T, tumor-bound trastuzumab-R27T was sufficient to produce cytotoxicity. However, the PBMC-mediated cytotoxic effects were not shown in Hs746T cells under wash-out condition (Figure 5C right). These results highlight the importance of providing IFN-β with a tumor-targeting property.

**FIGURE 6:**
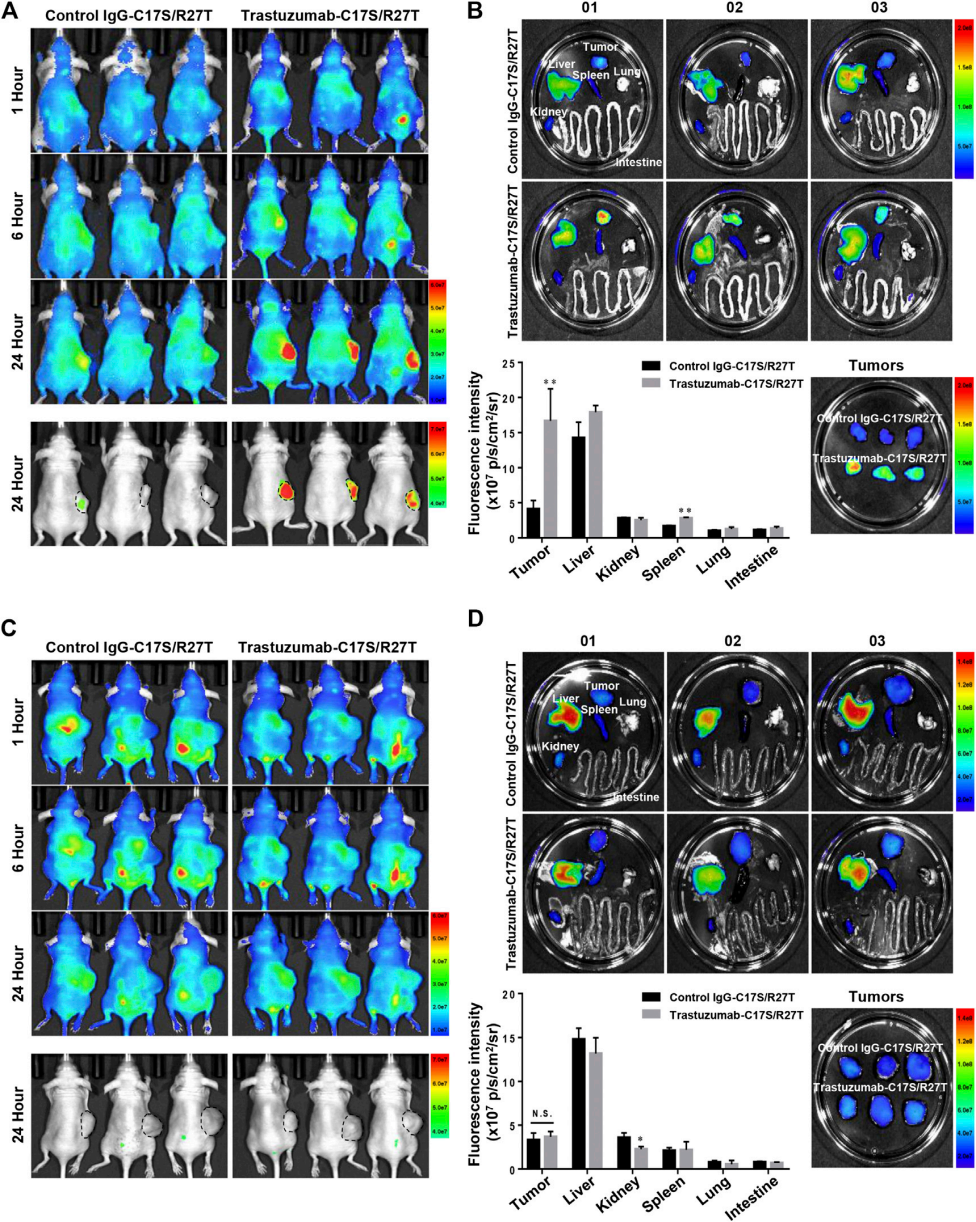
Tumor targeting effect of trastuzumab-IFN-β mutein in xenograft mice bearing HER2-positive or HER2-negative tumor. **(A**,**C)** NCI-N87 **(A)** or Hs746T **(C)** cells were transplanted subcutaneously into BALB/c-nude mice. CF750–control IgG-C17S/R27T or CF750–trastuzumab-C17S/R27T was intravenously injected at a dose of 100 μg/mouse, and the fluorescence intensity was monitored at 1, 6, and 24 h **(B**,**D)** Tissues from NCI-N87 **(B)** or Hs746T **(D)** xenograft mice were isolated at 24 h post-injection, and the *ex vivo* fluorescence images were observed. The *ex vivo* fluorescence images from each tissue were quantified as the average radiant efficiency. Data represent the mean ± SD (n = 3), **p < 0.01 vs. control IgG-C17S/R27T.

To demonstrate the *in vivo* tumor-targeting properties of trastuzumab-IFN-β mutein depend on whether HER2 is expressed or not, fluorescence imaging assay was conducted in xenograft mice bearing tumors with HER2 positive (NCI-N87) or negative (Hs746T). IgG-C17S/R27T, a fusion protein consisting of human IgG with IFN-β mutein as a non-targeting agent, and trastuzumab-C17S/R27T were conjugated with the fluorescence dye (CF750). Further, 100 µg of CF750-labeled control IgG-C17S/R27T or trastuzumab-C17S/R27T was intravenously injected in BALB/c nude mice bearing NCI-N87 or Hs746T to evaluate the tumor-targeting properties of these agents. On a qualitative basis, *in vivo* fluorescence imaging indicated that the fusion proteins were locally concentrated in the tumor and liver for 24 h after administration (Figures 6A,C). In addition, quantitative *ex vivo* measurements of the dissected organs of NCI-N87 tumor-bearing mice confirmed that trastuzumab-C17S/R27T targeted the HER2-positive tumor with more than four-fold high local concentration compared to control IgG-C17S/R27T (Figure 6B). In contrast to NCI-N87, there was no difference in tissue distribution, including in the tumor (Figure 6D). Collectively, the results demonstrated that trastuzumab-IFN-β-mutein was guided to the tumor in an HER2 dependent manner.

### *In vivo* Effect of Trastuzumab-IFN-β Mutein

**FIGURE 7:**
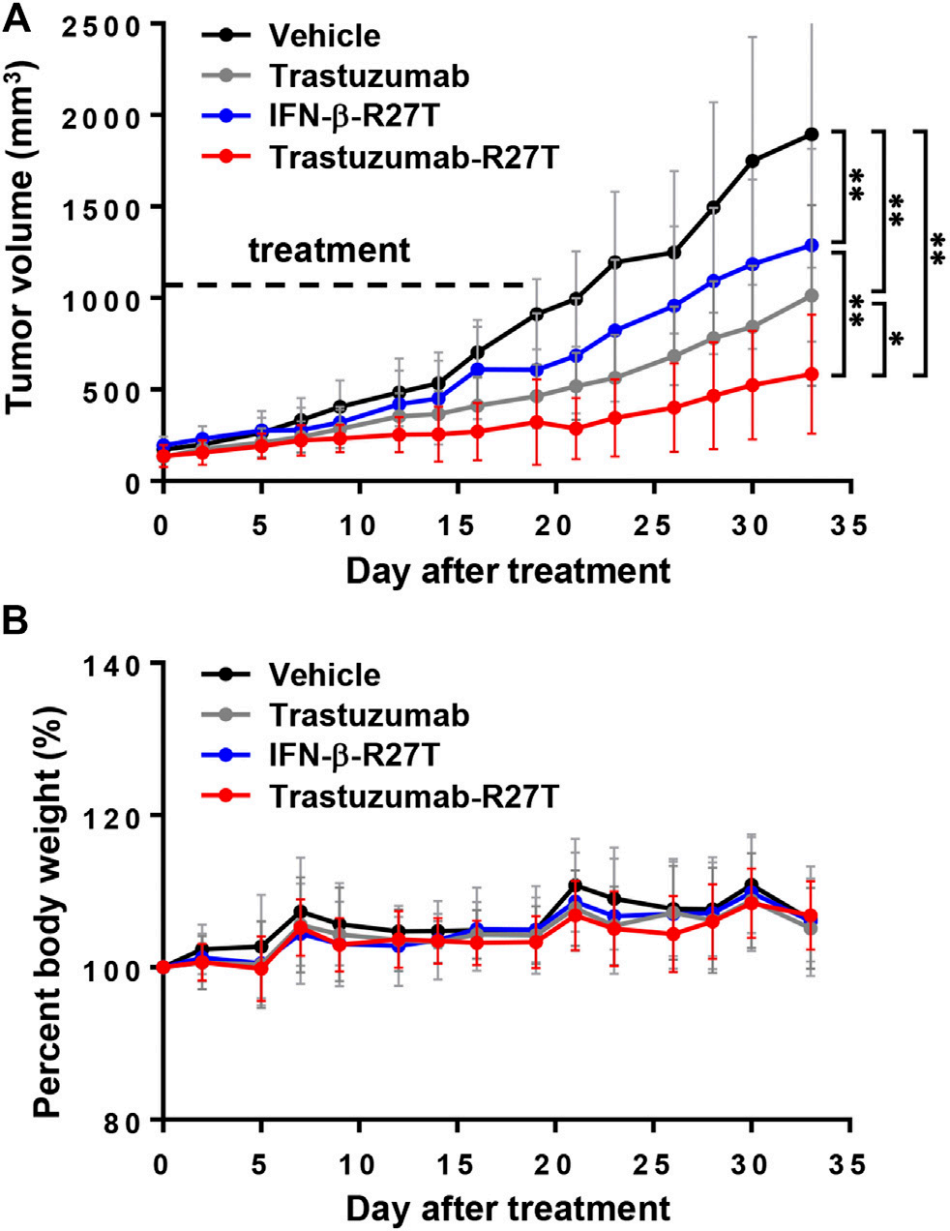
The *in vivo* direct effect of trastuzumab-IFN-β mutein in NCI-N87 xenograft model. **(A)** Tumor volume gain curve of mice treated with vehicle, trastuzumab, IFN-β-R27T, or trastuzumab-R27T (n = 4 per group). Dot line indicated the date of drug treatment. Tumor volume analysis was performed by two-way ANOVA. Data represent mean ± SD, *p < 0.05; **p < 0.01. **(B)** The percent of body weight gain curve.

The *in vivo* therapeutic performance of trastuzumab-IFN-β mutein was tested in BALB/c nude mice bearing NCI-N87 tumors. After the tumor volume reached the average volume of 150 mm^3^, the mice were treated with trastuzumab, IFN-β-R27T, or trastuzumab-R27T thrice a week for three weeks. Compared to vehicle control, trastuzumab, IFN-β-R27T, or trastuzumab-R27T inhibited the tumor growth effectively without a change in weight (Figures 7A,B). Although antitumor effect of trastuzumab-R27T is mostly similar to IFN-β-R27T *in vitro*, it was significantly higher than that of IFN-β-R27T *in vivo*. Because biophysical properties such as half-life and biodistribution are considerable factors for *in vivo* effect, the cytokine had no strong *in vivo* effects than the *in vitro* effects. However, trastuzumab-IFN-β mutein, which improved the biophysical properties of the cytokine and is endowed with tumor-targeting, exhibited superior efficacy over IFN-β-R27T.

**FIGURE 8:**
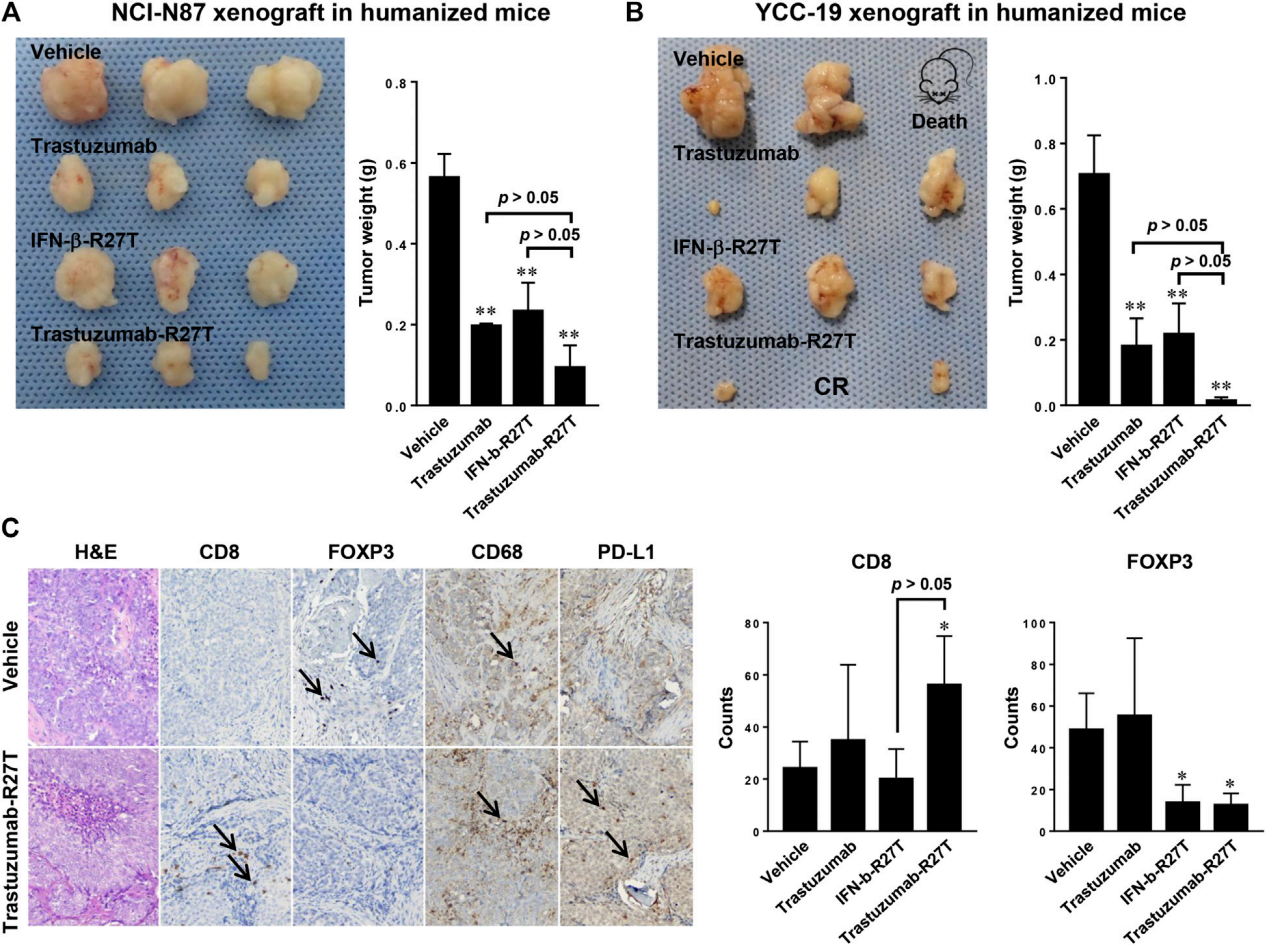
The *in vivo* effect of trastuzumab-IFN-β mutein in humanized gastric cancer xenograft models. **(A**,**B)** Tumor weight of humanized mice bearing NCI-N87 **(A)** or YCC-19 **(B)** treated with vehicle, trastuzumab, IFN-β-R27T, or trastuzumab-R27T (n = 3). Data represent mean ± SD, **p < 0.01 vs. vehicle. **(C)** Immunohistochemistry analysis of tumor-infiltrating immune cells after treatment. 10 μm sections of tumors were stained for CD8 (cytotoxic T cell), FOXP3 (regulatory T cell), CD68 (tumor-associated macrophage), and PD-L1 (immune check point). Magnification, ×100. Data represent mean ± SD, *p < 0.05, **p < 0.01 vs. vehicle.

The tumor regression effect of trastuzumab-R27T in the xenograft model only reflected the direct cancer cell growth inhibitory effects and did not include the immune cell-mediated antitumor effects. Because human IFN-β rarely responds to mouse cells (Harari et al., 2014), an *in vivo* model with a human immune system is required to reveal the immune responses. Hence, we developed CD34^+^ human hematopoietic stem cell-derived humanized mice, which recapitulated the human immune system (De La Rochere et al., 2018). Further, two humanized gastric cancer models were established using NCI-N87 and YCC-19. In both models, tumor size was significantly reduced in all treatment groups compared to vehicle control. Particularly, trastuzumab-R27T had greater efficiency than trastuzumab or IFN-β-R27T alone in both NCI-N87 and YCC-19 humanized xenograft tumor models (Figures 8A,B).

Immune cell infiltration to tumor tissue is necessary for the prognosis of patients and the response to immunotherapies (Fridman et al., 2017). So, we investigated the prevalence of tumor-infiltrating lymphocytes in NCI-N87 bearing humanized mice. The immunohistochemistry (IHC) analysis revealed that increased numbers of CD8^+^ T cells (CD8^+^) were observed in small tumors responding to trastuzumab-R27T therapy relative to controls (Figure 8C). The percentages of CD8^+^ T cells in the tumors of trastuzumab-R27T-treated mice were significantly higher than that of control tumors. In addition, type I IFNs negatively regulated the proliferation of regulatory T cells, which directly suppressed the activity of cytotoxic T cells (Pace et al., 2010; Sisirak et al., 2012). The number of regulator T cells (FOXP3+) was significantly decreased in the trastuzumab-R27T-treated group. All examined tumors exhibited very low numbers of monocyte (CD14^+^) cells and B cells (CD20^+^) in association with high macrophage (CD68^+^) numbers regardless of the treatment (data not shown and Figure 8C). PD-L1 was detected in tumor cells and stromal immune cells. The expressions of PD-L1 in tumor cells tended to be slightly increased by trastuzumab-R27T, which might be due to type I IFN-induced PD-L1 expression (Garcia-Diaz et al., 2017). Taken together, these data suggest that tumor repression by trastuzumab-IFN-β mutein relies on both direct and indirect immune cell-mediated cytotoxic activities.

## Discussion

This is the first study to describe the production of a novel immunocytokine comprising a human IFN-β, which selectively targets the HER2-positive tumor tissues, achieving a high local concentration of the cytokine at the site. In this study, we used IFN-β-R27T, a biobetter of IFN-β, to generate immunocytokine instead of wild-type IFN-β. We had developed the glycoengineered IFN- β-R27T mutant; the additional glycosylation makes it more physicochemically stable (Song et al., 2014). Several studies have revealed that undesirable physicohemical properties of IFN-β primarily cause the various types of its instability, such as aggregation, oxidation, deamidation, and disulfide changes. Therefore, IFN-β instability during the culture, purification, and storage of the commercial product has been investigated to decrease instability, especially aggregation, improve productivity and maintain functional activity (Rodriguez et al., 2005; Han et al., 2009; Rodriguez et al., 2010). IFN-β-R27T is less susceptible to biophysical instability, such as aggregation, than Wild-type IFN- β. Consequently, the productivity of IFN-β-R27T was approximately 3–6 times higher than that of wild-type IFN-β (Song et al., 2014). It is likely that this was a consequence of lesser aggregation resulting from the additional glycosylation, since it is well known that low productivity of IFN-β is caused by molecular aggregation. For immunocytokines, the stability of the proteins tends to be determined primarily by the cytokine rather than the antibody (Sommavilla et al., 2010; Schneider et al., 2019). This is because antibodies are very stable, whereas cytokines are relatively unstable, resulting in product-related impurities. The productivity of trastuzumab-R27T was higher than that of trastuzumab-wild-type IFN-β. This is probably because of the same reason why IFN-β-R27T showed up to 6-fold higher productivity than wild-type IFN-β.

The fusion protein potently inhibited human HER2-high gastric and breast cancers (Supplementary Figure S5) in direct manner. In contrast, HER2-low cancers showed differential response to IFN-β, even though STAT1 phosphorylation by IFN was observed (Figure 2B). Interestingly, the direct killing effect of trastuzumab-R27T is primarily dependent on IFN-β rather than trastuzumab in almost all cancer cell lines. However, its cytotoxic sensitivity to cytokines seems to be correlated with HER2 expression. Our results are in line with a previous clinical correlation study showed that IFN activity was associated with a low risk in patients with HER2-positive breast cancer (Callari et al., 2014) In addition, IFN could act as a regulatory factor against HER2-positive cancers (Stagg et al., 2011; Castiello et al., 2018). All these evidences indicate that the differences in the level of HER2 expression would be associated with distinct biological properties of cancer cells. To date, there has been no report on the effect of HER2 on the responsiveness to type I IFN. Therefore, the underlying mechanism of why higher IFN-induced cytolytic activity is observed in HER2-positive cancers needs to be studied in more depth in future research. The higher sensitivity of neoplastic cells with high HER2 expression to IFN may have clinical implications as it suggests the benefits of anti-HER2 conjugated IFN-β in patients with high-risk HER2-positive cancer.

The *in vitro* antitumor effect of trastuzumab-R27T is mostly dependent on IFN-β-R27T. However, the *in vivo* effect of trastuzumab-R27T was significantly greater than that of IFN-β-R27T (Figure 7). Unlike the *in vitro* situation, biophysical properties, such as half-life and biodistribution, are considerable factors for the effect of the drug *in vivo*. Despite the strong effect of IFN-β, no strong *in vivo* effects were observed resembling those *in vitro*. However, the immunocytokine, which improves the short half-life of cytokine and is endowed with tumor-targeting effects, retained its strong antitumor effect *in vivo* and *in vitro*. In general, the half-life of cytokine is short, ranging from several minutes to several hours. In the case of IFN-β, serum concentrations following intravenous administration decline rapidly, and the terminal half-life ranges from 1 to 2 h in humans (Wills, 1990). Therefore, most drugs are lost before they reach an optimal concentration in the target organs and have only a slight effect. Here, we fused trastuzumab to IFN-β mutein, which has a half-life of 15 h (unpublished data). Though its half-life is shorter than that of the antibody (terminal half-life of trastuzumab: 12–16 days) (Spilker et al., 2017), it causes a dramatic increase in half-life when compared to cytokine (terminal half-life of IFN-β-R27T: 88 min) (Song et al., 2014). Therefore, our immunocytokine is not merely the simple sum of a cytokine and an antibody, and it could be valuable as novel entity that has different biophysical properties than those of a cytokine or an antibody alone.

It is controversial whether the direct or indirect effect of IFNs is the main anticancer mechanism. In several studies, type I IFNs have direct inhibitory effects on tumor cells of different origin, hence, they curb proliferation and drive senescence and apoptosis. The immunocytokine, anti-CD20-IFN-α clearly showed that type I IFNs could have direct cytotoxic effects in lymphoma, which is very sensitive to type I IFN receptor-mediated tumor killing (Rossi et al., 2009; Xuan et al., 2010; Trinh et al., 2013). On the contrary, Yang at al. demonstrated that antibody-fused mouse IFN-β regulates tumor growth primarily by host (mouse) immune responses rather than direct killing (Yang et al., 2014). They provided evidence that the antibody fused mouse IFN-β enhanced cytotoxic T lymphocytes activation creating a positive feedback loop to kill more cells.

Given that the NCI-N87 cell line is IFN-sensitive, the *in vivo* direct tumor killing effect of trastuzumab-R27T in the xenograft model was consistent with the *in vitro* effect. Similarly, in our NCI-N87 and YCC-19 xenograft model using humanized mice, the direct antitumor effect of trastuzumab-R27T would also be effective. Furthermore, it was found that the number of tumor-infiltrating human CD8^+^ T cells increased in the trastuzumab-R27T-treated group in the humanized mice model. FOXP3 stained cells, presumed to be regulatory T cells were significantly decreased by a third compared to vehicle control. Rios-Doria et al. characterized several xenograft models using humanized mice and revealed that the antitumor response of the models to immunotherapy was positively associated with the induced levels of CD4 and CD8 tumor-infiltrating lymphocytes (Rios-Doria et al., 2020). Moreover, CD8 T cells were essential for the antitumor immune response of the murine IFN-β-immunocytokine (Yang et al., 2014). Based on these studies, our results showing the infiltration of CD8 T cells in humanized mice model provided a meaningful evidence for the immunological effects of human IFN-β immunocytokine. Collectively, previous reports and our results complement each other and cover two different modes of action for IFN-mediated tumor regression.

There are several limitations in the present study. Due to technical difficulties, we used tumor cell lines and did not freshly isolate autologous tumors to assess the cytotoxic capabilities of immune cells. Therefore, we mainly evaluated the antitumor activity of immune cells, except for the analysis of autologous tumor cells. Moreover, although we introduced humanized mice to verify the role of immune cells in antitumor effect, the direct anti-tumor effects were also mixed in the mice models, making it difficult to evaluate only intact indirect effects. In fact, the most important consideration of immunocytokine studies is the possibility of reducing cytokine toxicity through antigenic targeting. Unfortunately, it would be limited to evaluate the full efficacy and toxicity of human IFN-β substrates to host cells in murine models because human IFN-β rarely works in mouse cells (Harari et al., 2014). Therefore, the development of new models capable of evaluating intact human IFN-β is required, which may accelerate the translational research of human IFN-β.

In summary, the data presented in this study provide a rationale for the clinical investigation of fully human analogs of trastuzumab-IFN-β, which feature human IFN-β as the therapeutic payload. Targeting IFN-β with an anti-HER2 mAb makes the immunocytokine more potent than either agent alone. Our findings suggest that trastuzumab-IFN-β mutein merits clinical evaluation as a new candidate for anticancer therapeutics.

## Data Availability Statement

The datasets presented in this study can be found in online repositories. The names of the repository/repositories and accession numbers can be found in the article/Supplementary Material.

## Ethics Statement

The studies involving human participants were reviewed and approved by Seoul Metropolitan Government Seoul National University Boramae Medical Center and Seoul National University Institutional Review Board. The patients/participants provided their written informed consent to participate in this study. The animal study was reviewed and approved by Institutional Animal Care and Use Committee (IACUC) of Seoul National University.

## Author Contributions

CL, TK, and YS designed the study. CL, TK, SH, JC, KS, SR, HJ, and YS contributed to the conceptual development and experimental design. CL, TK, SH, JC, JK, HP, and HJ performed the experiments and analyzed the data. CL, SL, J-SC, and YS prepared the manuscript.

## Funding

This research was funded by the Global Core Research Center (Grant No. 2011-0030001) from the National Research Foundation, Ministry of Science and ICT, Korea; and Tech Incubator program for Startup Program (S2716361) funded by the Ministry of Small and Medium-sized Enterprises and Startups.

## Conflict of Interest

CL, JK, and HJ were employed by the company Genopharm Inc., and CL and HJ currently hold stock in Genopharm Inc., Korea. JC was employed by the company ABION Inc., and JC and YS currently hold stock in ABION Inc., Korea. SH and YS have received consulting fees from ABION Inc., Korea.

The remaining authors declare that the research was conducted in the absence of any commercial or financial relationships that could be construed as a potential conflict of interest.
