# Supplementary material for: Antibody-Based Targeting of Interferon-Beta-1a Mutein in HER2-Positive Cancer Enhances Antitumor Effects Through Immune Responses and Direct Cell Killing
Source: Front Pharmacol. 2021 Jan 8;11:608774. doi: 10.3389/fphar.2020.608774 (PMC7832035; doi:10.3389/fphar.2020.608774)
Supplement: Supplementary file 1 [file datasheet1.docx]

Supplementary Material

# Supplementary Materials and Methods

**IFN luciferase assay, flow cytometry analysis, and cell viability assay** All experimental methods were performed as described in the Materials and Methods section.

# Supplementary Figures and Tables

## Supplementary Figures


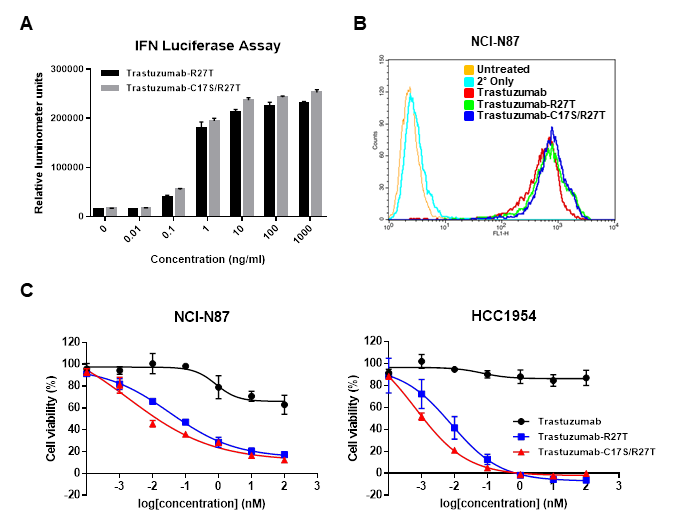


**Supplementary Figure S1.** Bioactivity of trastuzumab-C17S/R27T compared to trastuzumab-R27T. (A) IFN luciferase activity. (B) HER2 binding. (C) *In vitro* cell growth inhibitory effect.


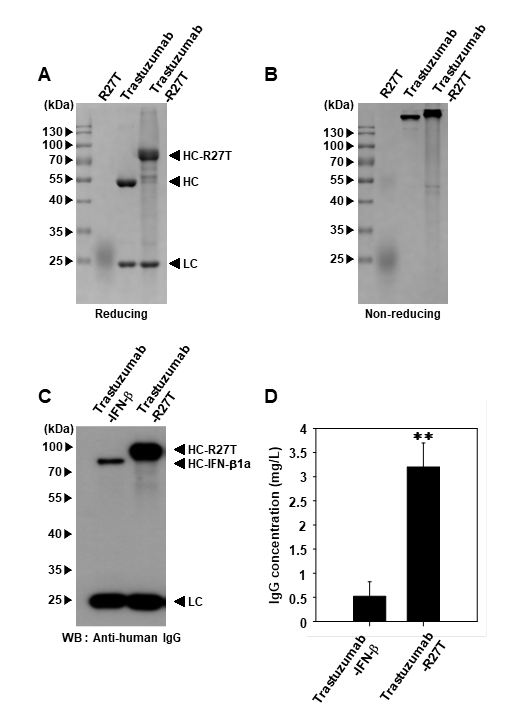


**Supplementary Figure S2.** Expression pattern of trastuzumab-R27T. Proteins were purified from culture fluids using Protein A columns and separated by SDS-PAGE under (A) reducing or (B) non-reducing conditions. Expression analysis was subjected to (C) western blot analysis with anti-human IgG (H+L) antibody to detect the heavy and light chains, or (D) IgG quantification using Cedex bio. Data represent the mean ± SD of three independent experiments (***p* < 0.01 versus trastuzumab-IFN-β).


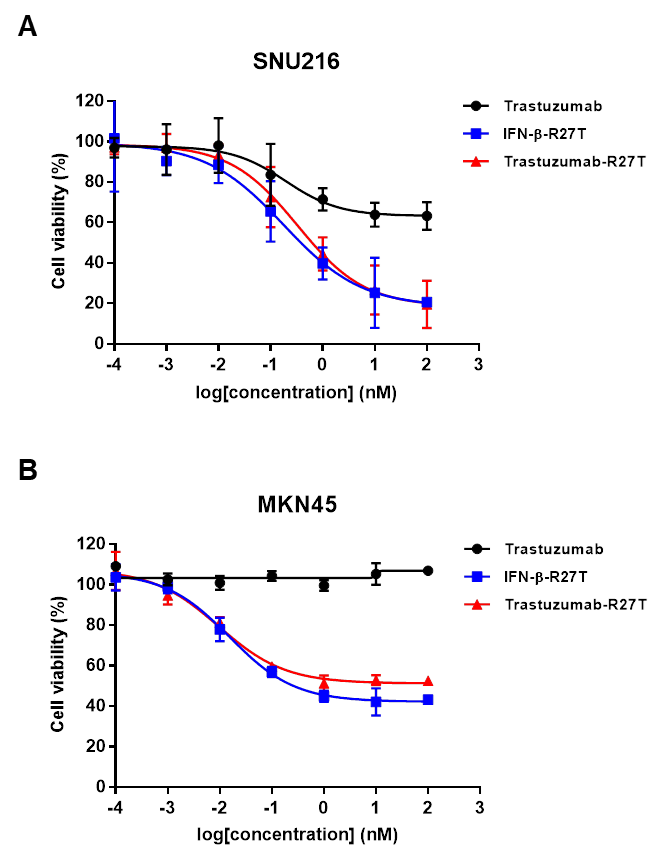


**Supplementary Figure S3.** *In vitro* direct antitumor effect of trastuzumab-R27T on SNU216 and MKN45 cells. SNU216 and MKN45 cells were treated with trastuzumab, IFN-β-R27T, or trastuzumab-R27T for 72 h. Cell growth (%) was determined using the WST assay.


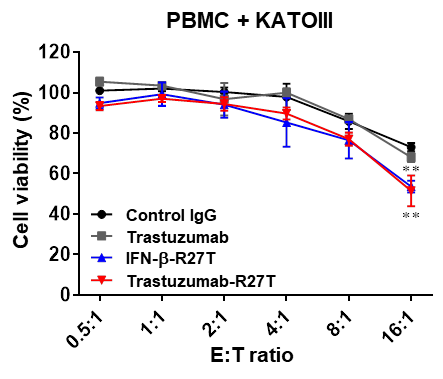


**Supplementary Figure S4.** *In vitro* PBMC-mediated antitumor effect of trastuzumab-R27T on KATOIII cells. PBMCs co-cultured with KATOIII cells were treated with 0.1 nM of control IgG, trastuzumab, IFN-β-R27T, or trastuzumab-R27T. At 72 h post treatment, cell viability was measured using the WST assay.


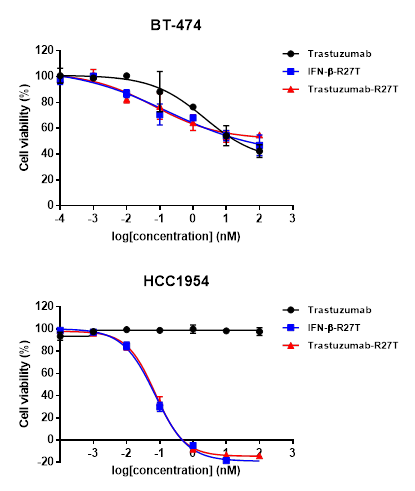


**Supplementary Figure S5.** *In vitro* direct antitumor effect of trastuzumab-R27T on breast cancer cell lines. BT-474 and HCC1954 cells were treated with trastuzumab, IFN-β-R27T, or trastuzumab-R27T for 72 h. Cell growth (%) was determined using the WST assay.

## Supplementary Table

**Supplementary Table 1.** Protein sequences of the antibody and antibody-fusion proteins

| Materials | Chain | Amino acid sequence (N to C) |
| --- | --- | --- |
| Trastuzumab | Heavy chain | EVQLVESGGGLVQPGGSLRLSCAASGFNIKDTYIHWVRQAPGKGLEWVARIYPTNGYTRYADSVKGRFTISADTSKNTAYLQMNSLRAEDTAVYYCSRWGGDGFYAMDYWGQGTLVTVSSASTKGPSVFPLAPSSKSTSGGTAALGCLVKDYFPEPVTVSWNSGALTSGVHTFPAVLQSSGLYSLSSVVTVPSSSLGTQTYICNVNHKPSNTKVDKKVEPKSCDKTHTCPPCPAPELLGGPSVFLFPPKPKDTLMISRTPEVTCVVVDVSHEDPEVKFNWYVDGVEVHNAKTKPREEQYNSTYRVVSVLTVLHQDWLNGKEYKCKVSNKALPAPIEKTISKAKGQPREPQVYTLPPSREEMTKNQVSLTCLVKGFYPSDIAVEWESNGQPENNYKTTPPVLDSDGSFFLYSKLTVDKSRWQQGNVFSCSVMHEALHNHYTQKSLSLSPGK |
|  | Light chain | DIQMTQSPSSLSASVGDRVTITCRASQDVNTAVAWYQQKPGKAPKLLIYSASFLYSGVPSRFSGSRSGTDFTLTISSLQPEDFATYYCQQHYTTPPTFGQGTKVEIKRTVAAPSVFIFPPSDEQLKSGTASVVCLLNNFYPREAKVQWKVDNALQSGNSQESVTEQDSKDSTYSLSSTLTLSKADYEKHKVYACEVTHQGLSSPVTKSFNRGEC |
| Tastuzumab  -wild-type IFN-β | Heavy chain | EVQLVESGGGLVQPGGSLRLSCAASGFNIKDTYIHWVRQAPGKGLEWVARIYPTNGYTRYADSVKGRFTISADTSKNTAYLQMNSLRAEDTAVYYCSRWGGDGFYAMDYWGQGTLVTVSSASTKGPSVFPLAPSSKSTSGGTAALGCLVKDYFPEPVTVSWNSGALTSGVHTFPAVLQSSGLYSLSSVVTVPSSSLGTQTYICNVNHKPSNTKVDKKVEPKSCDKTHTCPPCPAPELLGGPSVFLFPPKPKDTLMISRTPEVTCVVVDVSHEDPEVKFNWYVDGVEVHNAKTKPREEQYNSTYRVVSVLTVLHQDWLNGKEYKCKVSNKALPAPIEKTISKAKGQPREPQVYTLPPSREEMTKNQVSLTCLVKGFYPSDIAVEWESNGQPENNYKTTPPVLDSDGSFFLYSKLTVDKSRWQQGNVFSCSVMHEALHNHYTQKSLSLSPGKGGGGSGGGGSGGGSGMSYNLLGFLQRSSNFQCQKLLWQLNGRLEYCLKDRMNFDIPEEIKQLQQFQKEDAALTIYEMLQNIFAIFRQDSSSTGWNETIVENLLANVYHQINHLKTVLEEKLEKEDFTRGKLMSSLHLKRYYGRILHYLKAKEYSHCAWTIVRVEILRNFYFINRLTGYLRN |
|  | Light chain | DIQMTQSPSSLSASVGDRVTITCRASQDVNTAVAWYQQKPGKAPKLLIYSASFLYSGVPSRFSGSRSGTDFTLTISSLQPEDFATYYCQQHYTTPPTFGQGTKVEIKRTVAAPSVFIFPPSDEQLKSGTASVVCLLNNFYPREAKVQWKVDNALQSGNSQESVTEQDSKDSTYSLSSTLTLSKADYEKHKVYACEVTHQGLSSPVTKSFNRGEC |
| Trastuzumab  -R27T | Heavy chain | EVQLVESGGGLVQPGGSLRLSCAASGFNIKDTYIHWVRQAPGKGLEWVARIYPTNGYTRYADSVKGRFTISADTSKNTAYLQMNSLRAEDTAVYYCSRWGGDGFYAMDYWGQGTLVTVSSASTKGPSVFPLAPSSKSTSGGTAALGCLVKDYFPEPVTVSWNSGALTSGVHTFPAVLQSSGLYSLSSVVTVPSSSLGTQTYICNVNHKPSNTKVDKKVEPKSCDKTHTCPPCPAPELLGGPSVFLFPPKPKDTLMISRTPEVTCVVVDVSHEDPEVKFNWYVDGVEVHNAKTKPREEQYNSTYRVVSVLTVLHQDWLNGKEYKCKVSNKALPAPIEKTISKAKGQPREPQVYTLPPSREEMTKNQVSLTCLVKGFYPSDIAVEWESNGQPENNYKTTPPVLDSDGSFFLYSKLTVDKSRWQQGNVFSCSVMHEALHNHYTQKSLSLSPGKGGGGSGGGGSGGGSGMSYNLLGFLQRSSNFQCQKLLWQLNG**T**LEYCLKDRMNFDIPEEIKQLQQFQKEDAALTIYEMLQNIFAIFRQDSSSTGWNETIVENLLANVYHQINHLKTVLEEKLEKEDFTRGKLMSSLHLKRYYGRILHYLKAKEYSHCAWTIVRVEILRNFYFINRLTGYLRN |
|  | Light chain | DIQMTQSPSSLSASVGDRVTITCRASQDVNTAVAWYQQKPGKAPKLLIYSASFLYSGVPSRFSGSRSGTDFTLTISSLQPEDFATYYCQQHYTTPPTFGQGTKVEIKRTVAAPSVFIFPPSDEQLKSGTASVVCLLNNFYPREAKVQWKVDNALQSGNSQESVTEQDSKDSTYSLSSTLTLSKADYEKHKVYACEVTHQGLSSPVTKSFNRGEC |
